# Recent Chemical and Pharmacological Developments on 14-Oxygenated-*N*-methylmorphinan-6-ones

**DOI:** 10.3390/molecules26185677

**Published:** 2021-09-18

**Authors:** Mariana Spetea, Helmut Schmidhammer

**Affiliations:** Department of Pharmaceutical Chemistry, Institute of Pharmacy and Center for Molecular Biosciences Innsbruck (CMBI), University of Innsbruck, Innrain 80-82, 6020 Innsbruck, Austria; helmut.schmidhammer@uibk.ac.at

**Keywords:** morphine, morphinans, 14-alkoxymorphinans, opioid agonist, multifunctional opioid ligands, drug design, structure–activity relationships, pain, antinociception, side effects

## Abstract

Adequate pain management, particularly chronic pain, remains a major challenge associated with modern-day medicine. Current pharmacotherapy offers unsatisfactory long-term solutions due to serious side effects related to the chronic administration of analgesic drugs. Morphine and structurally related derivatives (e.g., oxycodone, oxymorphone, buprenorphine) are highly effective opioid analgesics, mediating their effects via the activation of opioid receptors, with the mu-opioid receptor subtype as the primary molecular target. However, they also cause addiction and overdose deaths, which has led to a global opioid crisis in the last decades. Therefore, research efforts are needed to overcome the limitations of present pain therapies with the aim to improve treatment efficacy and to reduce complications. This review presents recent chemical and pharmacological advances on 14-oxygenated-*N*-methylmorphinan-6-ones, in the search of safer pain therapeutics. We focus on drug design strategies and structure–activity relationships on specific modifications in positions 5, 6, 14 and 17 on the morphinan skeleton, with the goal of aiding the discovery of opioid analgesics with more favorable pharmacological properties, potent analgesia and fewer undesirable effects. Targeted molecular modifications on the morphinan scaffold can afford novel opioids as bi- or multifunctional ligands targeting multiple opioid receptors, as attractive alternatives to mu-opioid receptor selective analgesics.

## 1. Introduction

Pain, particularly severe and chronic pain, constitutes a major public health problem with an enormous impact on both the individual and society. Successful pain management can be viewed as a combination of adequate analgesia and minimal unwanted side effects. However, currently available analgesics are either ineffective in a large proportion of patients or the benefit/risk ratio is suboptimal due to multiple and severe adverse effects [1,2]. Pain medicine is currently one of the most rapidly developing medical specialties, with effective pain control being a therapeutic priority [3,4]. Furthermore, comorbidity of chronic pain with mood (e.g., depression and anxiety) and addictive disorders (e.g., alcohol and drug abuse) in pain patients is well-documented [4,5,6].

Strong opioids are the mainstay for the treatment of moderate to severe nociceptive pain, such as post-operative pain and pain due to major trauma. They are only third-line treatments for neuropathic pain [1,7]. Over the past decades, there has been an increased focus on pain management, which has resulted in a societal escalation in opioid use and misuse, with opioids being presently the most prescribed medications in the USA [2]. As a consequence, a dramatic growth in the number of opioid-related overdose deaths and diagnoses of opioid-use disorder associated with prescription and over-the-counter opioids was experienced in recent years [8,9]. Therefore, pain medicine and analgesic drug discovery are intensively dedicated to the discovery of effective, safer and nonaddictive analgesic drugs [9,10]. Diverse strategies are being evaluated to mitigate the deleterious side effects of opioids, ranging from abuse-deterrent formulations of existing opioids to peripherally restricted opioids, and from multifunctional drugs to biased agonists. A comprehensive review on such developments is beyond the scope of this mini-review, and we recommend extended and topical reviews and books [11,12,13,14,15,16,17,18,19,20,21].

The therapeutic analgesia of opioid drugs is mediated by the opioid receptors [22,23]. To date, four opioid receptors have been cloned, i.e., the mu (MOP), delta (DOP), kappa (KOP) and nociceptin (NOP) receptors. All opioid receptor types are G protein-coupled receptors (GPCRs) with seven transmembrane domains, which bind endogenous opioid ligands [22,23]. A key milestone in the field was realized with the high-resolution crystal structures of all opioid receptors attained in active and inactive conformations [24,25,26,27,28,29,30]. Furthermore, the present understanding of the opioid system’s function and signaling, together with significant information on the structural basis for the pharmacological profile of endogenous and exogenous ligands, is constantly growing with the crystal structures of the receptors being nowadays available [31,32].

**Figure 1:**
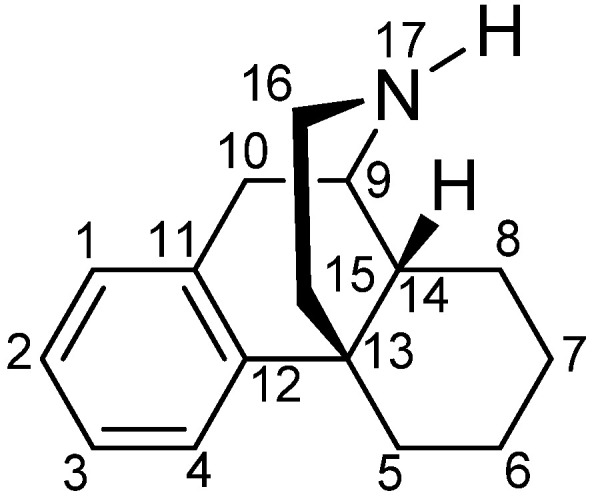
The (−)-morphinan skeleton.

**Figure 2:**
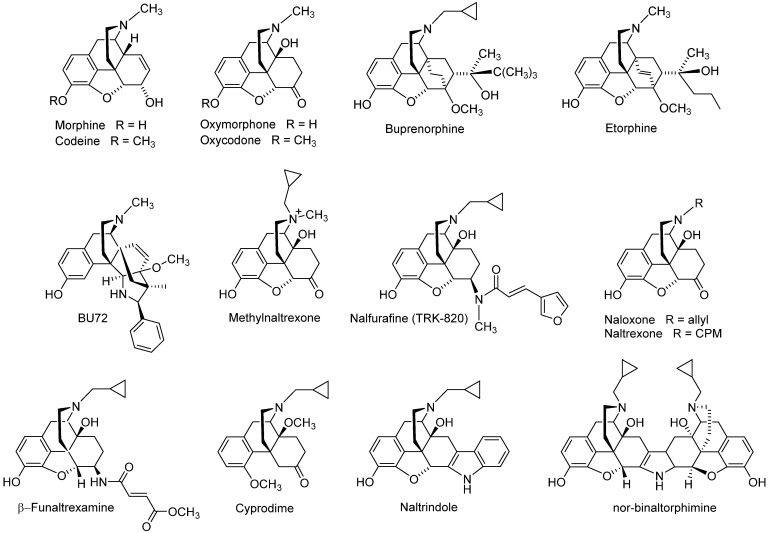
Examples of opioid morphinans used in the clinics or as research tools. CPM, cyclopropylmethyl.

Historically, the MOP receptor has been of most clinical interest because it mediates the desired analgesic action of opioids, but also their detrimental effects, which include respiratory depression, constipation, sedation and, with prolonged treatment, tolerance, dependence and abuse liability [7,22,23,33]. The MOP receptor is the endogenous target of naturally occurring peptides, while it is also responsible for the analgesic effect of morphine, structurally derived compounds and other opioid drugs. Morphine, the natural alkaloid from the poppy plant, *Papaver somniferum*, has been used for decades for pain relief, and its addictive and other side effects are well-known [34]. Since its structure elucidation in 1925 by Gulland and Robinson [35] and first synthesis of morphine in 1956 by Gates and Tschudi [36], research has been constantly focused on finding a non-narcotic opioid drug. The skeleton of morphine is a nitrogen-containing phenanthrene derivative belonging to the class of morphinans (Figure 1). One of the first ‘nonaddictive morphine substitutes’ to be introduced was the 3,6-diacetylated derivative, diacetylmorphine (heroin), but the claims for reduced respiratory depression and dependence liability were soon shown to be ill-founded [37]. Numerous chemical efforts have contributed to the design and synthesis of various derivatives of morphine that are used as therapeutics and/or important research tools (Figure 2), with references to comprehensive literature reviewed over the years [38,39,40,41,42,43,44,45,46,47,48,49,50].

Clinically used opioid analgesics from the class of morphinans comprise naturally occurring alkaloids (e.g., morphine, codeine), semi-synthetic derivatives (e.g., oxycodone, oxymorphone, buprenorphine) and synthetic analogues (e.g., levorphanol, butorphanol, etorphine) (Figure 2). Such drugs proved to be of the utmost significance for the treatment of moderate to severe nociceptive pain as a result of their binding and agonist action at the MOR, while they all share the same general pharmacological profile, including undesired side effects [51,52]. A KOP receptor agonist from the class of morphinans, nalfurafine (Figure 2) [53], was originally developed as an analgesic drug for post-operative pain, but the safety margin was found to be insufficient for analgesic use [54]. Because of nalfurafine’s anti-scratching/anti-itching effects in animals and humans, it is clinically used in Japan for the treatment of uremic pruritus in individuals with chronic kidney disease undergoing hemodialysis [55,56].

Development of opioid antagonists with a morphinan structure was also reported, exemplified by the non-selective antagonists naloxone and naltrexone, the MOP receptor selective antagonists β-funaltrexamine and cyprodime, the DOP receptor selective antagonists naltrindole and the KOP receptor selective antagonist nor-binaltophimine (Figure 2) [41,48,57,58]. Whereas antagonists were initially employed as pharmacological tools in opioid research, there has been attention in generating selective antagonists as potential pharmacotherapies for the treatment of human disorders where the opioid system (opioid receptors and their ligands) plays an important role. For example, naloxone and naltrexone are in clinical practice as treatments for narcotic overdose and alcoholism or opioid abuse/dependence [48,57,59]. The quaternary analogue of naltrexone, methylnaltrexone (Figure 2), a peripheral MOP antagonist, is used as a medication for the treatment of opioid-induced constipation [60]. The MOP selective antagonist β-funaltrexamine and agonist BU-72 (Figure 2) were used to elucidate the X-ray crystal structures of the MOP receptor in inactive [25] and active conformations [28], respectively, whereas the crystal inactive structure of the DOP receptor bound to the antagonist, naltrindole (Figure 2) was reported [24].

This review summarizes recent chemical and pharmacological developments in the area of opioid analgesics through exploring the morphinan scaffold in the search of safer pain therapeutics. We particularly focus on the structure–activity (SAR) relationships of differently substituted 14-oxygenated morphinans, including design strategies, synthetical procedures and pharmacology.

## 2. Modifications in Position 6 of 14-Oxygenated-*N*-methylmorphinan-6-ones: Design, Synthesis and SAR Studies

Among the clinically used opioid analgesics, *N*-methylmorphinan-6-ones, such as oxycodone and oxymorphone (Figure 2), are of major value in pain treatment, although they also produce severe side effects [7,48,51]. Particularly, the licit and illicit use of prescription opioid painkillers, particularly oxycodone, has led to a global opioid crisis, with thousands of addiction cases and overdose deaths during the past years [8,9]. Functionalization and modification of the C-6 carbonyl group of morphinans has been persistently the focus of medicinal chemistry towards the discovery of efficacious and safer opioids. The chemically highly versatile 6-keto function of mophinan-6-ones permits conversion into ligands with a large diversity of biological activities [61,62,63,64,65]. Representative and recent examples on such synthetic approaches leading to chemically innovative structures and pharmacologically interesting opioid ligands are presented herein.

### 2.1. Deletion of the Carbonyl Group in Position 6 via Wolff–Kishner Reduction

**Scheme 1:**
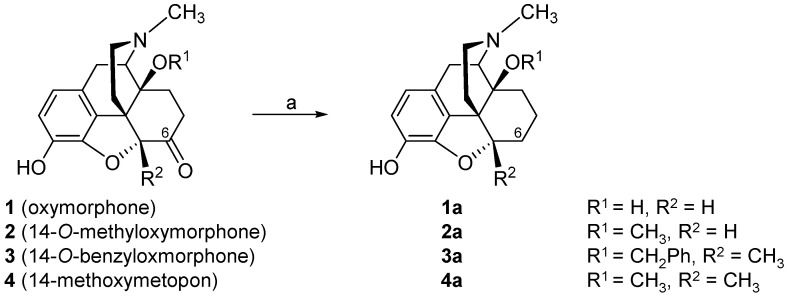
Structures of *N*-methylmorphinan-6-ones **1**–**4** and their 6-desoxo counterparts **1a**–**4a**, and their preparation via the *Wolff*–*Kishner* reduction. Reagents and conditions: (**a**) hydrazine hydrate, triethylene glycol, 180 °C, 1.5 h, then KOH pellets, 2 h. Ph, phenyl.

The consequence of the deletion of the 6-carbonyl group of 14-hydroxy (oxymorphone **1**) and 14-alkoxy substituted *N*-methylmorphinan-6-ones **2**–**4** (Scheme 1) leading to the respective 6-desoxo-*N*-methylmorphinans **1a**–**4a**, on in vitro opioid activities and antinociceptive potencies, was described [66]. The *Wolff–Kishner* reduction [67,68] was applied for the preparation of compounds **1a**–**4a** starting from their corresponding 6-keto analogues.

Binding in vitro studies with Chinese hamster ovary (CHO) cells expressing the human opioid receptors showed that in the series of *N*-methylmorphinan-6-ones **1**–**4**, the removal of the 6-keto function did not considerably affect binding affinity and agonist potency at the MOP receptor (**1** vs. **1a**, **2** vs. **2a** and **4** vs. **4a**), with the exception of **3** vs. **3a**, where a 9-fold increase in the inhibition constant (K_i_) value at the MOP receptor and about 2.5-fold in the potency (EC_50_) at the MOP receptor were reported for the *N*-methyl-14-benzyloxy substituted **3a** (Table 1) [66]. Generally, the 6-desoxo analogues displayed similar binding affinities at the DOP receptor to their 6-ketomorphinan counterparts, the exception being oxymorphone (**1**) and its analogue 14-*O*-methyloxymorphine (**1a**), where a 3-fold decrease in the DOP affinity was observed for **1a**. Affinities at the human KOP receptor of the 6-desoxo derivatives **1a**–**4a** were in the range of their *N*-methylmorphinan-6-ones **1**–**4**. In terms of MOP receptor selectivity, the deletion of the 6-carbonyl group in oxymorphone (**1**) improved selectivity for the MOP vs. DOP receptor of its 6-desoxo analogue **1a**, without changing MOP vs. KOP receptor selectivity. In the case of the 14-methoxy substituted **2** and **2a**, a minor reduction in the MOP vs. KOP receptor selectivity was found, while selectivity for the MOP vs. DOP receptor was not altered. A larger reduction in the selectivity for MOP vs. DOP receptor and vs. KOP receptor was produced by the deletion of the 6-keto group in the 5-methyl substituted **4**. An interesting SAR observation was made for the nonselective 14-*O*-benzyloxymorphone **3**, being converted into an MOP receptor selective ligand, **3a**, showing about 9- and 6-fold higher selectivity than **3** for MOP vs. DOP and MOP vs. KOP, respectively (Table 1) [66]. Pharmacological in vivo studies also reported on the effect of the deletion of the 6-carbonyl group of 14-hydroxy and 14-alkoxy substituted *N*-methylmorphinan-6-ones **1**–**4** on the antinociceptive activity in a mouse model of acute thermal nociception (the hot-plate assay) after subcutaneous (s.c.) administration [66]. It was described that the 6-desoxo compounds **1a**–**4a** exhibit comparable antinociceptive potencies to their analogue **1**–**4** (Table 1), demonstrating that the absence of a 6-carbonyl group does not influence the in vivo agonism. The side effect profiles of the 6-desoxo-*N*-methylmorphinans were not yet reported.

Because of its therapeutic significance, the MOP receptor is among the few GPCRs determined in different activation states, with the first X-ray crystal structure of the receptor protein bound to β-funaltrexamine (PDB code: 4DKL) [25], and the 3D structure in the active conformation where the receptor was co-crystallized with the agonist BU72 (PDB code: 5C1M) [28]. Recently, the cryo-electron microscopy (cryo-EM) structures of the MOP receptor (PDB codes: 6DDE and 6DDF) bound to the agonist peptide [*D*-Ala^2^,*N*-Me-Phe^4^,Gly-ol^5^]enkephalin DAMGO were described [69]. To appreciate the result of the 6-carbonyl group deletion in *N*-methylmorphinan-6-ones on pharmacological in vitro profiles, a molecular docking study using the active conformation of the MOP receptor was reported [66]. At the in silico level, the absence of the 6-carbonyl function in **3a** depleted the steric clash shown by the 6-keto group of **3** with the Val^300^ residue, offering a rationale for the improved MOP receptor binding affinity of **3a**, as experimentally determined in radioligand binding assays.

### 2.2. Introduction of Acrylonitrile Substructures in Position 6

Earlier studies reported on different synthetical approaches for converting the C-6 carbonyl group in 14-oxygenated-*N*-methylmorphinan-6-ones (i.e., oxycodone, oxymorphone, dihydromorphinone) into a variety of functionalities, for example hydrazones, oximes, semicarbazone, amino, guanidine, sulfate and amino acid derivatives [64,65,70,71,72,73,74,75,76,77]. Subsequent pharmacological studies showed that such modifications generally do not affect the opioid activities of the ligand, with the resulted compounds being potent antinociceptive agents in rodents with reduced unwanted side effects (i.e., respiratory depression and constipation) [61,64,65,72,73,74,78,79].

**Scheme 2:**
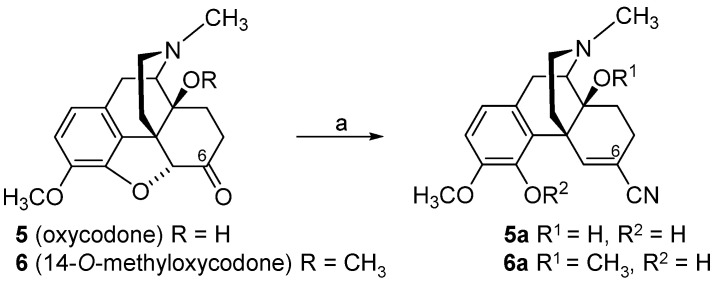
Synthetic route to 4,5-epoxy-ring opened 5,6-didehydromorphinan-6-carbonitriles, **5a** and **6a**. Starting from oxycodone (**5**) and 14-*O*-methyloxycodone (**6**), the expected carbonitriles were not obtained. Instead, the ether bridge between position 4 and 5 of the starting compound was opened and the respective acrylonitrile derivatives **5a** and **6a** were formed exclusively. Reagents and conditions: (**a**) TosMIC, potassium *tert*-butoxide (*t*-BuOK), dimethyl ether (DME), *tert*-butanol (*t*-BuOH), 0 °C.

**Scheme 3:**
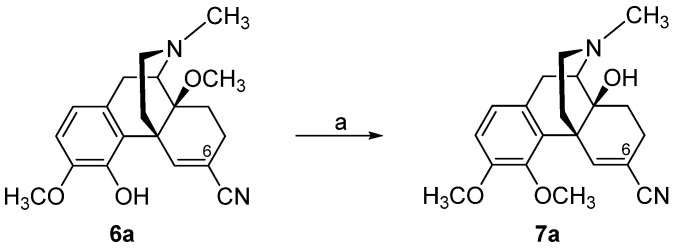
Synthetic route to 4,5-epoxy-ring opened 5,6-didehydromorphinan-6-carbonitrile **7a**. Reagents and conditions: (**a**) A mixture of **6a**, potassium carbonate, phenyltrimethylammonium chloride and dimethylformamide (DMF) was stirred at 80 °C under N_2_ for 5 h.

By targeting the chemically highly versatile 6-keto function of *N*-methylmorphinan-6-ones as in oxycodone (**5**), we reported on a chemically innovative modification leading to a new class of morphinans with acrylonitrile incorporated substructures [80,81,82]. An interesting strategy to include acrylonitrile substructures into morphinans is represented by the van Leusen homologation reaction [83], wherein tosylmethylisocyanid (TosMIC) reacts with carbonyl compounds to provide the corresponding nitriles with one additional carbon atom. Using a modified van Leusen reaction, the 4,5-oxygen bridge in oxycodone (**5**) and 14-*O*-methyloxycodone (**6**) and the introduction of a 6-cyano group were achieved concurrently in a convenient, high-yield one-pot reaction, resulting in 4,5-epoxy-ring opened acrylonitrile derivatives **5a** and **6a**, respectively (Scheme 2) [80]. Additionally, the synthesis of the 4-*O*-methylated derivative of **5a**, namely **7a**, was reported (Scheme 3) [81].

**Scheme 4:**
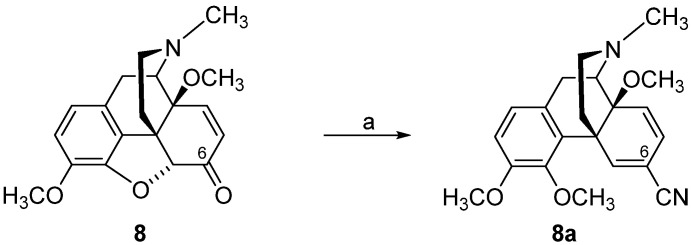
Synthetic route to compound **8a**. Reagents and conditions: (**a**) Compound **8** was added to a cooled (0 °C) suspension of NaH in 15 mL anhydrous DMF, and the mixture was stirred for 30 min, then CH_3_I was added.

**Scheme 5:**
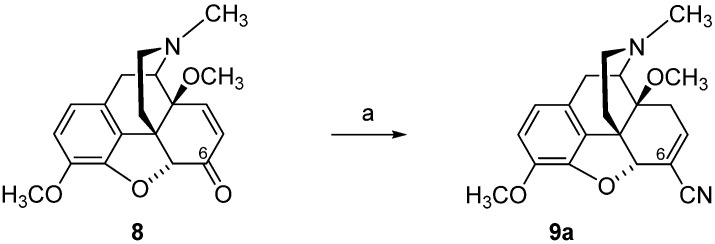
Synthetic route to 6,7-didehydrocarbonitrile **9a**. Reagents and conditions: (**a**) TosMIC, *t*-BuOK, DME, *t*-BuOH, 0 °C.

As reported in [82], TosMIC converted 7,8-didehydro-6-ketomorphinan (**8**) into 5,6,7,8-tetrahydromorphinan-6-carbonitrile **8a** (Scheme 4). Opposite to the cyanation of oxycodone (**5**) and 14-*O*-methyloxycodone (**6**) with TosMIC, 7,8-didehydromorphinan-6-ones, such as 14-methoxycodeinone (**8**), afforded 6,7-didehydrocarbonitrile **9a**, with retainment of the 4,5-epoxy ring under these conditions (Scheme 5) [82].

SAR studies explored how and to what degree the presence of a 6-cyano group in *N*-methylmorphinans affected opioid receptor binding and activation profiles, and antinociceptive activities compared to the 6-keto analogues [81,84]. The first chemical and pharmacological study on acrylonitrile incorporated 4,5-oxygen bridge opened *N*-methylmorphinans [81] reported that a 6-cyano substituent leads to MOP receptor selective morphinan ligands (**5a**–**7a**) with high MOP receptor affinity and reduced binding at the other opioid receptor subtypes, DOP and KOP receptors, in rodent brain tissues (Table 2). In vivo, the 6-cyanomorphinans **5a**–**7a** were highly effective against thermal (hot-plate and tail-flick tests) and chemical nociception (paraphenylquinone (PPQ)-induced writhing test) in mice after s.c. administration, with increased antinociceptive potency than oxycodone (**5**) and 14-*O*-methyloxycodone (**6**) (Table 2). The presence of a 14-methoxy substituent (**6a**) instead of a 14-hydoxy group (**5a**) in 6-cyanomorphinans not only increases in vitro MOP receptor binding affinity, but also enhances the antinociceptive potency (Table 2 and Table 3) [81].

The initial attractive strategy to incorporate acrylonitrile substructures into morphinans was further investigated and resulted in two 14-methoxy-*N*-methylmorphinas **8a** and **9a** (Scheme 4 and Scheme 5) [82]. The design of analogue **8a** with a 4,14-dimethoxy substitution was achieved based on the observation that a 4-methoxy group and/or a 14-methoxy group, such as in **6a** and **7a**, is more favorable for binding and selectivity for the MOP receptor and antinociceptive activity than the corresponding hydroxyl counterpart **5a** (Scheme 2) [81,84]. The presence of a methoxy group in both positions, 4 and 14, had a major, positive impact on the binding affinities to all three opioid receptor types, while preserving MOP receptor selectivity, but also on MOP receptor agonist potencies and efficacies (Table 2 and Table 3) [84]. Additionally, it was investigated how the combination of a C-6 cyano functionality together with a closed 4,5-oxygen bridge (**9a**) influences in vitro and in vivo opioid activities [84]. The two 6-cyanomorphinans **6a** and **9a** show comparable, low nanomolar affinities at the MOP receptor and potent MOP agonism (Table 2). In vivo, the 6-cyanomorphinan **9a** was more active than its 4,5-oxygen bridge-opened analogue **6a** in inducing an antinociceptive effect in mice after s.c. administration. Closing of the 4,5-oxygen bridge in the 6-acrylonitrile-substituted **6a** produces no major alterations in interaction with the MOP receptor in vitro, but improved antinociceptive potency (Table 3). Early behavioral observations in mice receiving 6-cyanomorphinas reported the absence of sedative effects at antinociceptive doses, with only a minor increase in locomotor activity seen at doses higher than the antinociceptive ED_50_ doses [84].

## 3. Modifications in Position 14 of 14-Oxygenated-*N*-methylmorphinan-6-ones: Design, Synthesis and SAR Studies

The 14-position of natural opioids (e.g., morphine and codeine, see Figure 2) are unsubstituted; however, synthetic approaches have uncovered that functionalizing position 14 gives rise to a broad array of activities. Diverse modifications in position 14 of the morphinan skeleton were targeted by us and others with the prospect of designing novel effective opioid analgesics, with fewer unwanted adverse effects [43,44,45,46,47,48,85,86,87]. We have reported on the substitution of the hydroxyl group in position 14 in the clinically used MOP agonist oxymorphone (**1**) with a methoxy group leading to 14-*O*-methyloxymorphone (**2**, Scheme 1) [88]. Introduction of a 14-methoxy group in oxymorphone (**1**) not only increased binding affinity and agonist potency at the MOP receptor, but also caused a significant improvement in the antinociceptive activity of 14-*O*-methyloxymorphone (**2**) in different pain models in rats and mice (for reviews, see [47,48]). It was reported to exhibit about 40-fold higher antinociceptive potency than oxymorphone and was up to 400-fold more effective that the “gold standard” morphine. Unfortunately, 14-*O*-methyloxymorphone (**2**) induced the typical MOP-mediated side effects, including respiratory depression, physical dependence, constipation and motor dysfunction [88,89,90,91].

Using 14-*O*-methyloxymorphone (**2**) as a lead compound, numerous series of differently substituted 14-alkoxymorphinan-6-ones were designed and their pharmacology was reported (for reviews, see [44,46,47,48]). A notable example is 14-*O*-benzyloxymorphone (**3**), where the 14-methoxy group in **2** was replaced by a benzyloxy group [89]. 14-*O*-Benzyloxymorphone (**3**) retained the high affinity in the subnanomolar at the MOP receptor of **2**, while having increased MOP agonist potency in vitro and in vivo (Table 1 and Table 4). Furthermore, antinociceptive potency of derivative **3** was reported as 5-fold higher compared to **2** (Table 1 and Table 5), in addition to its limited inhibition of gastrointestinal motility in mice at antinociceptive doses. It exhibited 2.5-fold less constipation than morphine and was 7-fold less effective than **2** in this respect [89].

Using the crystal structure of the MOP receptor in the active conformation (PDB code: 5C1M) [28], the first molecular modeling study aided by docking and molecular dynamics (MD) simulations reported on the binding modes and interaction pattern differences related to specific structural features of oxymorphone (**1**) and 14-*O*-methyl and 14-*O*-benzyl substituted analogues **2** and **3**, respectively [92]. Similar to oxymorphone (**1**), derivatives **2** and **3** bind at the MOP receptor and form a charged interaction with Asp^147^ and a hydrogen bond to His^297^ via a water network [92]. The later interaction is recognized as a conserved interaction between morphinan ligands and the binding pocket of the MOP receptor [25,28,66,93,94]. The presence of the 14-methoxy group in **2** does not change the ligand–receptor interaction profile compared to **1**, as the polar contacts of **2** were comparable to those of **1** except for the hydrogen bond formed by the 14-hydroxyl group of **1** to the Asp^147^ residue. Interesting were the docking results on the 14-benzyloxy substituted derivative **3**, where the intermolecular hydrogen bonds were maintained, established by the comparison of the binding mode of this analogue to the docking solution of 14-methoxy analogue **2**. An additional hydrophobic interaction to Ile^144^ residue was described for **3** [92].

**Scheme 6:**
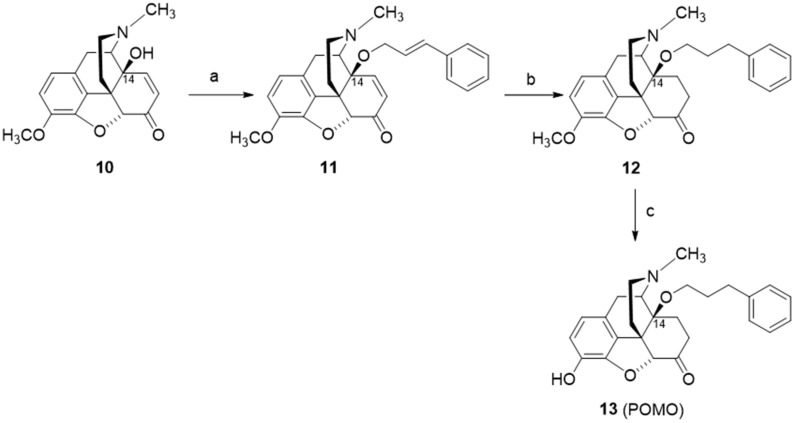
Synthetic route to POMO (**13**). Reagents and conditions: (**a**) cinnamyl bromide, NaH, DMF, 0 °C; (**b**) hydrogenation over Pd/C, glacial acetic acid, r.t., 40 psi; (**c**) 3-*O*-ether cleavage, refluxing 48% HBr solution.

The design and synthesis of the 14-*O*-phenylpropyl substituted analog of 14-*O*-methyloxymorphone (**2**), namely 14-*O*-phenylpropyloxymorphone (POMO, **13**) (Scheme 6), as an interesting representative of the series of *N*-methyl-14-arylalkoxy-morphinan-6-ones, was described [91,95]. Starting from 14-hydroxycodeinone (**10**), POMO was prepared in three synthetic steps (Scheme 6) [95].

Recent pharmacological investigations reported POMO (**13**) as a potent mixed MOP/DOP/KOP receptor agonist with reduced propensity for constipation in mice after s.c. administration [91]. An interesting SAR observation was revealed where replacing the 14-*O*-methyl in **2** by a 14-*O*-phenylpropyl moiety in **13** converted an MOP receptor selective ligand into a nonselective agonist (Table 4). In vivo, POMO (**13**) was highly effective in acute thermal nociception (hot-plate test) in mice after s.c. administration, with over 70- and 9000-fold increased potency than 14-*O*-methyloxymorphone (**2**) and morphine, respectively, while producing a four-fold lower inhibition of the gastrointestinal transit than **2** and morphine (Table 5) [91]. The pharmacological profile established for POMO (**13**), as a ligand that can simultaneously bind and activate multiple opioid receptors, is of major relevance nowadays, with the design of bi- and multifunctional opioids becoming increasingly attractive as novel strategy for an effective and safer pain management [16,18,85,87,96,97,98,99].

Other SAR observations were made in 4,5α-epoxypyridomorphinans, where the addition of a 3-phenylpropoxy substituent at C-14 on the framework containing a cyclopropylmethyl group on the morphinan nitrogen led to ligands with an MOP agonist/DOP antagonist profile, whereas *N*-methyl-14-*O*-phenylpropyl substituted analogues were reported as dual MOP/DOP agonists [85]. Recently, the MOP/DOP agonist SRI-22141 was described to have efficacy with reduced tolerance and dependence in mouse models of neuropathic pain [98].

## 4. Modifications in Position 5 of 14-Oxygenated-*N*-methylmorphinan-6-ones: Design, Synthesis and SAR Studies

Further chemical and synthetical efforts focused on structural modifications in position 5 of 14-methoxy-*N*-methylmorphinan-6-ones with evolving compounds showing an interesting pharmacological profile, as potent antinociceptives with an improved side effect profile [47,100]. Chemical derivatization of 14-*O*-methyloxymorphone (**2**) by introducing a methyl group in position 5 created 14-methoxymetopon (**4**) [101]. This new opioid morphinan retained the high subnanomolar affinity at the MOP receptor, shown by its 5-unsubstituted analogue **2**, while DOP and KOP receptor affinities were reduced by two- to three-fold, causing an increase in the MOP receptor selectivity (Table 6). It was also established that methylation in position 5 of **2** did not substantially alter MOP receptor agonist activity in vitro and in vivo. In the [^35^S]GTPγS binding assays with CHO cells expressing human opioid receptors (Table 6) [94] and in the rat brain [90], 14-methoxymetopon (**4**) showed comparable MOP receptor agonist potency and equal efficacy to its analogue **2** (Table 6). In vivo, the addition of a 5-methyl substituent in 14-*O*-methyloxymorphone (**2**) was well-tolerated, leading to a highly potent MOP analgesic **4**, which was very effective in reducing pain response in various thermal, chemical and inflammatory pain models in mice, rats and dogs, after different routes of administration (Table 7) (for a review, see [100]). Numerous behavioral studies reported on the significantly reduced propensity of 14-methoxymotopon (**4**) to induce MOP-mediated unwanted liabilities (i.e., respiratory depression, hypotension, bradycardia, constipation, analgesic tolerance, physical dependence, addiction) in animals (rodents and dogs) in comparison to conventional MOP analgesics, such as morphine and sufentanil (for a review, see [100]).

**Scheme 7:**
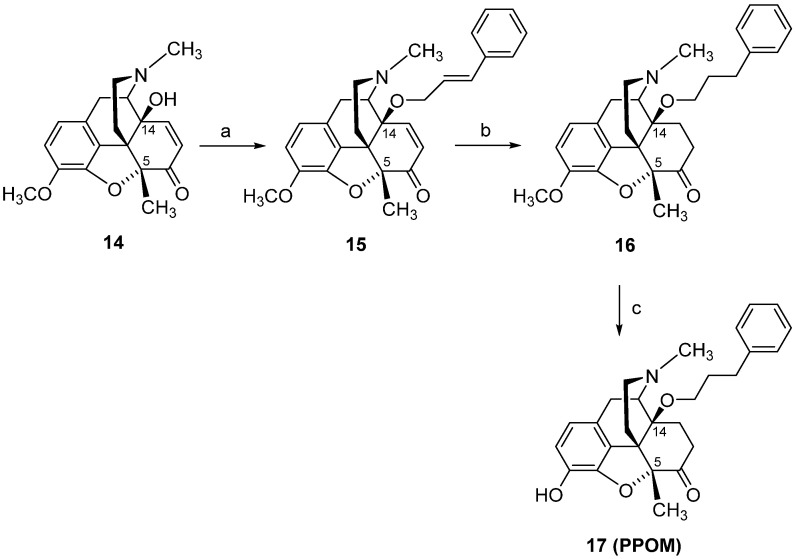
Synthetic route to PPOM (**17**). Reagents and conditions: (**a**) a mixture of **14**, cinnamyl bromide, DMF, NaH, 0 °C; (**b**) a mixture of **15**, EtOH, Pd/C, was hydrogenated at 50 °C for 2 h; (**c**) a mixture of **16** and 48% HBr was refluxed for 20 min, and after cooling to r.t., the mixture was treated with MeOH and evaporated to dryness.

**Scheme 8:**
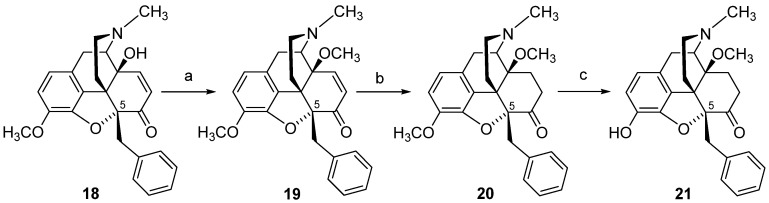
Synthetic route to 14-methoxy, 5-benzyl substituted **21**. Reaction scheme for the synthesis of 5-benzyl substituted morphinans. Reagents and conditions: (**a**) NaH, (CH_3_O)_2_SO_2_, DMF, 0 °C; (**b**) 10% Pd/C, H_2_, MeOH, 30 psi, r.t.; (**c**) 48% HBr, reflux.

In view of the remarkable in vitro and in vivo functional profile of 14-methoxymetopon (**4**), our interest in the field of opioid analgesics from the class of 14-alkoxymorphinan-6-ones was directed towards the design of analogues of **4**. Two representative derivatives, including the 14-phenylpropoxy, 5-methyl substituted PPOM (**17**) (Scheme 7) [102] and the 14-methoxy-5-benzyl substituted **21** (Scheme 8) [90], are described herein. Starting from 14-hydroxy-5-methylcodeinone (**14**) [103], PPOM (**17**) was prepared in three synthetic steps (Scheme 7). The 5-benzyl substituted analogue **21** was also prepared in three synthetic steps from 5ß-benzyl-14-methoxycodeinone (**19**), which is readily available from 5β-benzylthebaine (**18**) (Scheme 8) [104].

The phenylpropoxy substitution in position 14 of 14-methoxymetopon (**4**) led to a new structure **17**, with significant enhanced binding affinities at both DOP and KOP receptors, in the subnanomolar range, while the high MOP receptor affinity remained unchanged compared to **4**, with the result in a complete loss of MOP receptor selectivity for PPOM (**17**) (Table 6) [102]. Noteworthy are the behavioral findings in different pain models (hot-plate, tail-flick and writhing tests) in mice with PPOM (**17**), evolving as an extremely potent agonist in vivo with a considerably improved antinociceptive potency compared to its 14-methoxy substituted **4** (up to 400-fold) and morphine (up to 24,000-fold) (Table 7). PPOM (**17**) also showed a superior antinociceptive activity compared to etorphine (Figure 2) (up to 25-fold) [102], an MOP agonist used for anesthesia in veterinary medicine of large animals and wildlife species [105]. Such SAR observation on conversion of a selective MOP ligand into a multifunctional ligand, which displays activity at multiple opioid receptor subtypes, is very exciting in the light of the current research on analgesic drug discovery [16,18,96,97].

The SAR outcome on the substitution of the 5-methyl group in 14-methoxymetopon (**4**) by a benzyl group resulting in analogue **21** was investigated in order to understand the role of the substitution pattern in position 5 in *N*-methylmorphinan-6-ones on the opioid activity in vitro and in vivo [90]. In vitro, 5-benzyl substituted **21** showed a similar binding profile to the opioid receptors in the rodent brain to its 5-methyl analogue **4**. Replacing the 5-methyl group in **4** by a benzyl group in **21** kept the high affinity in the subnanomolar range at the MOP receptor and the MOP selectivity (Table 6). In the [^35^S]GTPγS binding assays with membranes from the rat brain and CHO cells expressing human opioid receptors, the presence of a 5-benzyl group in **21** yielded a potent MOP full agonist with a 5-fold increased potency than its 5-methyl analogue **4** at the rat MOP receptor and similar potency at the human MOP receptor (Table 6). A comparable in vitro MOP agonism was observed between the 5-benzyl analogue **21** and the 5-unsubstituted derivative 14-*O*-methyloxymorphone (**2**) (Table 6). Pharmacological in vivo studies in mice also demonstrated that the substitution of the 5-methyl group in **4** by a benzyl group was well-tolerated, leading to a highly potent and efficacious MOP antinociceptive agent **21 [90]**. Antinociceptive potency of **21** in mouse models of acute thermal nociception (hot-plate and tail-flick tests) after s.c. administration was comparable to that of 14-methoxymetopon (**4**), whereas a significantly increased potency to morphine was reported (Table 7) [90]. Additional behavioral studies evaluated the consequences of the replacement of the 5-methyl group in **4** by the 5-benzyl group in **21** on motor coordination in mice using the rotarod assay. Whereas 14-methoxymetopon (**4**), as well as its 5-unsubstituted analogue **2** and morphine, caused a significant motor deficit, the 5-benzyl substituted **21** did not affect the evoked locomotor activity of mice at doses producing a full antinociceptive efficacy [90].

The importance of the substitution pattern in position 5 to the binding mode of differently 5-substituted *N*-methylmorphinan-6 ones (**2**, **4**, **17** and **21**) to the active conformation of the human MOP receptor (PDB code: 5C1M) [28] was examined by molecular docking and MD stimulations [92]. A 5-methyl group introduced during targeted derivatization resulted in enhanced hydrophobic ligand-receptor interactions, as the orientation of these derivatives relative to the receptor was influenced by this structural modification. It was also proposed that this might influence the orientation of further groups (i.e., at position 14), predicted to interact favorably with the MOP receptor. Notable were the in silico findings on the 14-phenylpropoxy-5-methyl substituted analogue POMO (**17**). The in vitro and in vivo activity profiles of **17**, as a high affinity MOP agonist and one of the most potent opioid analgesic drug (Table 6 and Table 7), were supported by docking of the ligand to the active MOP receptor. Compared to other derivatives, an additional hydrophobic region was seen for **17**, embedding the phenyl group in this region formed by hydrophobic or aromatic residues (i.e., Trp^133^, Val^143^ and Ile^144^) [92]. In the SAR exploration on how the nature of the substituent at position 5 affects the ligand–MOP receptor interaction profile, the 5-H (**2**), 5-methyl (**4**) and 5-benzyl (**21**)-substituted derivatives were directly compared. Although all ligands exhibit similar MOP binding affinities (Table 6) and polar interactions with Asp^147^ and with His^297^ residues via a water network, other important ligand–receptor contacts were different or unexpected for **21** [92]. It was reported that **21**, as a potent MOP agonist, interacts with Asp^147^, albeit with reduced proneness, while the polar contact to the nearby Tyr^326^ residue was formed rather frequently, which is a unique characteristic of **21**. In MD stimulations, the 5-benzyl substituted **21** shows a remarkable tendency to form a hydrogen bond to Tyr^148^ residue, by either the 3-phenol group, the 4,5α-epoxy group or both. It was also observed that the basic nitrogen reorients in an unexpected manner, moving toward Tyr^326^ and away from the conserved Asp^147^, the counterpart in the charge-enhanced hydrogen bond, which is (more or less) common among the other morphinans [92].

## 5. Modifications in Position 17 of *N*-Methylmorphinan-6-ones: Design, Synthesis and SAR Studies

Over many years of research on morphine and structurally related derivatives, SAR analyses have assigned an important role to the N-substituent on the morphinan skeleton in defining the functional activity profile, extending from agonism to mixed agonism–antagonism to antagonism [38,44]. Historically, exchanging the *N*-methyl group in morphine by an allyl group generated the first compound to be reported as an opioid antagonist, nalorphine [38]. Nalorphine, also known as *N*-allylmorphine, was synthesized from morphine in 1942 [106]. Clinical studies showed that nalorphine reversed the analgesic and respiratory depressant effects of morphine and other narcotic analgesics, and precipitated the abstinence syndrome in morphine-dependent subjects [42,43,47]. Nalorphine acts as an antagonist at the MOP receptor and a partial agonist to full agonist at the KOP receptor, being the first drug used for narcotic overdose treatment. However, it exhibits undesirable psychotomimetic side effects, such as dysphoria and visual hallucinations due to its KOP activity; therefore, it is no longer used medically [43]. Further substitutions at the N-17 position were targeted with numerous compounds synthesized and pharmacologically characterized. In the 1960s, synthetical work on the replacement of the *N*-methyl group in oxymorphone by *N*-cycloproplymethyl or *N*-allyl groups led to the discovery of naloxone and naltrexone (Figure 2) [39,107]. They are well-known, non-selective opioid antagonists used as therapeutic agents (for the treatment of opioid overdose and drug and alcohol dependence) and important research tools in opioid receptors pharmacology [47,59,108].

**Figure 3:**
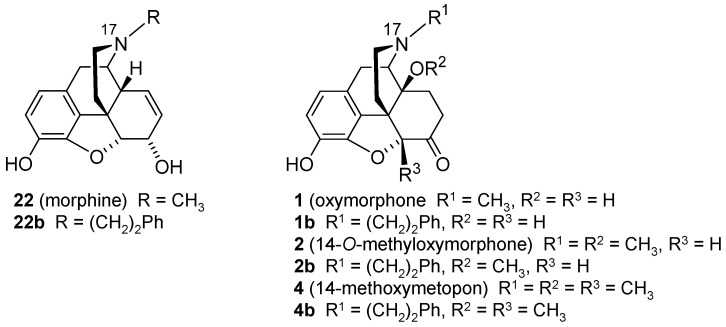
Structures of *N*-methylmorphinans morphine (**22**), oxymorphone (**1**), 14-*O*-methyoxymorphone (**2**) and 14-methoxymetopon (**4**), and their *N*-phenethyl counterparts **22b**, **1b**, **2b** and **4b**, respectively. Ph, phenyl.

Replacement of the *N*-methyl group with a different substituent, the *N*-phenethyl moiety, was also shown to influence the in vitro and in vivo functional profile of the opioid ligand. Among the first synthesized *N*-phenethyl substituted compounds were *N*-phenethylnormorphine [109,110], *N*-phenethylmorphine (**22b**, Figure 3) [111], *N*-phenethylnoroxymorphone [112] and *N*-phenethyloxymorphone (**1b**, Figure 3) [113], which were found to be potent MOP agonists and effective antinociceptive agents with increased potency compared to their *N*-methyl analogues [94,114,115,116]. Furthermore, exchanging the *N*-methyl group by an *N*-phenethyl substituent in other opioid ligands, such as 5-phenylmorphans, led to MOP antagonists [117,118]. A recent study reported on (-)-*N*-phenethyl analogs of *N*-norhydromorphone, with the *N*-*p*-chlorophenethylnorhydromorphone as a bifunctional MOP/DOP ligand with partial agonism at the MOP receptor and a full agonism at the DOP receptor, and antinociceptive efficacy without respiratory depression in squirrel monkeys after s.c. administration [119].

**Scheme 9:**
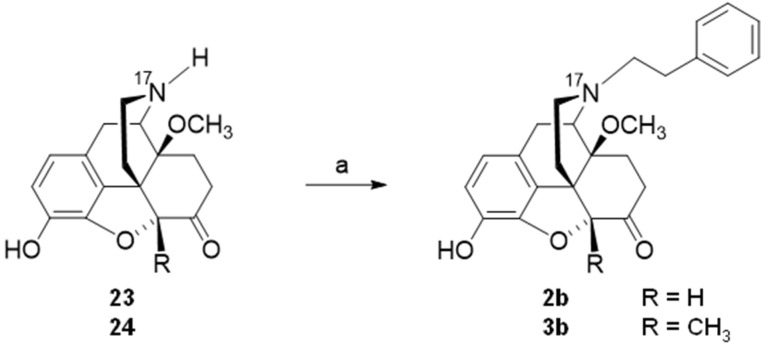
Synthetic route to *N*-phenethyl, 14-methoxy substituted **2b** and **3b**. Reagents and conditions: (**a**) phenethyl bromide, NaHCO_3_, DMF, 80 °C, 48 h.

The design of two *N*-phenethyl substituted 14-methoxymorphinan-6-ones was recently reported [94]. Based on the interesting pharmacological profile shown by 14-*O*-methyloxymorphone (**2**) and 14-methoxymetopon (**4**), it was aimed at investigating the effect of the replacement of the *N*-methyl group in **2** and **4**, by an *N*-phenethyl group in **2b** and **4b**, respectively, on binding and agonist in vitro activity at the opioid receptors and in vivo behavioral pharmacology [94]. Starting from **23** (4,5α-epoxy-3-hydroxy-14-methoxy-*N*-phenethylmorphinan-6-one) or **24** (4,5α-epoxy-3-hydroxy-14-methoxy-5-methyl-*N*-phenethylmorphinan-6-one), compounds **2b** and **3b**, respectively, were prepared in one synthetic step (Scheme 9) [94].

Remarkable SAR observations were made for *N*-methyl substituted **2** and **4** and their *N*-phenethyl substituted counterparts **2b** and **4b**, and also extended to the *N*-phenethyl analogues of morphine and oxymorphone, **22b** and **1b**, respectively (Figure 3) [94]. Whereas substitution of the *N*-methyl group in morphine (**22b**) and oxymorphone (**1**) by an *N*-phenethyl group enhances binding affinity, selectivity and agonist potency at the MOP receptor of **22b** and **1b**, the *N*-phenethyl substitution in 14-methoxy-*N*-methylmorphinan-6-ones (**2** and **4**) converts selective MOP ligands into dual MOP/DOP agonists (**2b** and **4b**) (Table 8 and Table 9). On the other hand, the high antinociceptive potency of *N*-methyl-14-methoxy substituted **2** and **4** in mice after s.c. administration was not affected by the presence of an *N*-phenethyl substituent in **2b** and **4b**, with both compounds being very effective antinociceptive agents against acute thermal nociception (Table 9) [94]. In contrast, the replacement of the *N*-methyl group in morphine (**22**) and oxymorphone (**1**) by an *N*-phenethyl moiety produced a significant increase in antinociceptive potencies of analogues **22b** and **1b** (Table 9) [94,114,115,116]. Behavioral studies also demonstrated that the *N*-phenethyl substituted **2b** and **4b**, with a dual MOP/DOP agonism, did not impair motor function of mice in the rotarod test at doses equivalent to the 4-fold the antinociceptive ED_50_ dose [94]. As noted earlier in this review, currently, there is a large interest in the discovery of multifunctional ligands, including bifunctional MOP/DOP ligands, expected to produce fewer undesirable side effects caused by selective MOP agonists [16,18,96,97,98].

Pharmacological findings in the series of *N*-phenethyl substituted **22b**, **1b**, **2b** and **4b** and their *N*-methyl analogues morphine (**22**), oxymorphone (**1**), 14-*O*-methyoxymorphone (**2**) and 14-methoxymetopon (**4**) were supported by docking and MD simulations analysis using the active conformation of the MOP receptor (PDB code: 5C1M) [28]. The recent report [94] established that 14-methoxy substituted *N*-methylmorphinan-6-one and their *N*-phenethyl counterparts share several essential receptor–ligand interactions, but also interaction pattern differences related to specific structural features. The *N*-methyl substituted morphine (**22**) and oxymorphone (**1**) showed a slightly different orientation in the binding pocket of the MOP receptor compared to their related *N*-phenethyl analogues, **22b** and **1b**, respectively. This was not observed for pairs **2** vs. **2b** and **4** vs. **4b**, indicating that an increased MOP receptor affinity can be achieved by either a 14-methoxy or by an *N*-phenethyl substitution, as key sites to be targeted in modulating the binding affinity and efficacy of morphinans at the MOP receptor [94].

## 6. Conclusions

Effective, safer and nonaddictive opioid analgesics have been continuously sought since the structural elucidation of morphine, characterization of multiple opioid receptor types and identification of endogenous opioid ligands. It has been the mission of scientists to design, synthesize and pharmacologically characterize novel lead molecules and to identify new mechanism-based treatment strategies, in order to find for an alternative to morphine that would produce strong analgesia but without its undesirable side effects, particularly abuse liability. Increased research efforts in the analgesic drug discovery are also justified by the large number of patients suffering of chronic pain conditions and the global, 21st-century opioid epidemic.

In this review, we have spotlighted recent advances in medicinal chemistry and pharmacology of new opioid ligands from the class of 14-oxygenated-*N*-methylmorphinan-6-ones towards the discovery of novel opioid analgesics with fewer unwanted side effects compared to currently available pain therapeutics. We discussed diverse chemical strategies combined with pharmacological work and molecular docking and MD simulations, and the emerged ligand-based SARs on specific modifications in positions 5, 6, 14 and 17 on the morphinan skeleton, thus offering a better understanding on how such structural variations influence the in vitro binding and in vivo opioid functional profiles. Notable were the findings on tuning the pharmacological properties in this class of ligands by turning selective MOP receptor ligands into multifunctional ligands with activity at multiple opioid receptor subtypes (MOP/DOP/KOP or MOP/DOP). Such developments in the identification of novel, innovative opioid ligands represent a persistent aspect in the discovery of opioid analgesics devoid of adverse effects for a superior pain management.

## Figures and Tables

**Table 1:** In vitro and in vivo opioid activity of *N*-methylmorphinan-6-ones **1**–**4** and their 6-desoxo counterparts **1a**–**4a**.

	Binding Affinity (K_i_, nM) ^a^				MOP Agonist Activity ^b^		Antinociception ^c^
	MOP	DOP	KOP	K_i_ Ratios MOP/DOP/KOP	EC_50_ (nM)	% stim.	ED_50_ (µg/kg, s.c.)
**1**	1.79	70.0	25.3	1/39/14	7.80	91.5	382
**1a**	1.65	201	21.6	1/1122/13	8.99	104	383
**2**	0.32	8.80	10.1	1/28/32	1.45	96.4	14.2
**2a**	0.24	8.64	3.99	1/36/17	1.37	105	17.3
**3**	0.45	1.05	1.27	1/2.3/1.8	0.32	101	2.38
**3a**	0.048	0.89	0.85	1/21/18	0.13	103	2.41
**4**	0.25	19.8	15.1	1/79/60	3.28	99.2	27.1
**4a**	0.34	12.9	7.92	1/38/23	2.95	104	25.1

^a^ Determined in competition radioligand binding assays using membranes of CHO cells stably expressing the human opioid receptors. ^b^ Determined in the [^35^S]GTPγS binding assay using CHO-hMOP cell membranes; percentage stimulation relative to the standard MOP receptor agonist DAMGO. ^c^ Determined in the hot-plate assay in mice after s.c. administration. Data from [66]. For compound structures, refer to Scheme 1.

**Table 2:** In vitro opioid activities of *N*-methylmorphinan-6-ones **5** and **6**, and their 6-CN substituted analogues **5a**–**9a**.

	Binding Affinity (K_i_, nM) ^a^				MOP Agonist Activity ^b^	
	MOP	DOP	KOP	K_i_ Ratios MOP/DOP/KOP	EC_50_ (nM)	% stim.
**5**	43.6	1087	2658	1/25/61	500	92
**5a**	31.7	498	1648	1/16/52	273	98
**6**	35.3	116	454	1/3/13	325	137
**6a**	5.38	197	378	1/37/70	26.2	85
**7a**	2.44	107	364	1/44/149	42.5	97
**8a**	0.54	30.3	200	1/56/370	1.64	133
**9a**	7.39	239	194	1/32/26	25.1	121

^a^ Determined in competition radioligand binding assays using rat brain membranes. ^b^ Determined in the [^35^S]GTPγS binding assay using CHO-hMOP cell membranes; percentage stimulation relative to the standard MOR agonist DAMGO. Data from [81,84]. For compound structures, refer to Scheme 3, Scheme 4 and Scheme 5.

**Table 3:** Antinociceptive activities of *N*-methylmorphinan-6-ones **5** and **6** and their 6-CN substituted analogues **5a**–**9a**.

	ED_50_ (mg/kg, s.c.) ^a^		
	Hot-Plate Test	Tail-Flick Test	PPQ Abdominal Stretching Test
**5**	1.37	0.94	0.38
**5a**	0.50	1.88	0.18
**6**	1.02	0.80	0.22
**6a**	0.25	0.21	0.11
**7a**	0.15	0.12	0.026
**8a**	0.080	0.040	0.0023
**9a**	0.089	0.12	0.003

^a^ Determined in mice after s.c. administration. Data from [81,84]. For compound structures, refer to Scheme 3, Scheme 4 and Scheme 5.

**Table 4:** Binding affinities and agonist potencies of *N*-methylmorphinan-6-ones **2**, **3** and **13**.

	Binding Affinity (K_i_, nM) ^a^				Agonist Activity (EC_50_, nM; %stim) ^b^		
	MOP	DOP	KOP	K_i_ ratios MOP/DOP/KOP	MOP	DOP	KOP
**2**	0.10	4.80	10.2	1/48/102	1.62; 97	43.8; 106	144; 65
**3**	0.12	2.14	1.18	1/18/10	0.32; 101	- ^c^	-
**13**	0.073	0.13	0.30	1/1.8/4.1	0.082; 100	0.28; 91	0.38; 39

^a^ Determined in competition radioligand binding assays using membranes from rat brain (MOP and DOP receptor) or guinea pig brain (KOP receptor). ^b^ Determined in the [^35^S]GTPγS binding assay using CHO stably expressing human opioid receptors; percentage of stimulation relative to the standard full agonists DAMGO (MOP), DPDPE (DOP) or U69,593 (KOP). ^c^ not determined. Data from [66,89,91]. For compound structures, refer to Scheme 1 and Scheme 6.

**Table 5:** Antinociceptive activities and gastrointestinal transit inhibition by *N*-methylmorphinan-6-ones **2**, **3** and **13** and morphine.

	Antinociception ^a^ (AD_50_ nmol/kg, s.c.)	Gastrointestinal Transit ^b^ (EC_50_ nmol/kg, s.c.)
**2**	53	37
**3**	9.6	27 ^c^
**13**	0.70	1.70
Morphine	6690	3800 7622 ^c^

^a^ Determined in the hot-plate test in mice after s.c. administration. ^b^ Determined in the charcoal test in mice after s.c. administration. ^c^ Determined in the colonic bead expulsion test in mice after s.c. administration. Data from [89,91]. For compound structures, refer to Scheme 1 and Scheme 6.

**Table 6:** Binding affinities and agonist potencies of differently 5-substituted *N*-methylmorphinan-6-ones **2**, **4**, **17** and **21**.

	Binding Affinity (K_i_, nM) ^a^				Agonist Activity (EC_50,_ nM; %stim) ^b^		
	MOP	DOP	KOP	K_i_ Ratios MOP/DOP/KOP	MOP	DOP	KOP
**2**	0.10	4.8	10.2	1/48/102	1.21; 95 23.7; 103 ^d^	38.5; 102	135; 65.6
**4**	0.15	13.3	25.2	1/89/168	2.66; 99 63.0; 99 ^d^	36.8; 100	181; 68.9
**17**	0.20	0.14	0.40	1/0.7/2	- ^c^	-	-
**21**	0.31	13.1	22.8	1/42/73	1.86; 95 ^e^ 13.8; 85 ^d^	31.7; 126 ^e^	116; 59.0 ^e^

^a^ Determined in competition radioligand binding assays using membranes from rat brain (MOP and DOP receptors) or guinea pig brain (KOP receptors). ^b^ Determined in the [^35^S]GTPγS binding assays using CHO stably expressing human opioid receptors; percentage stimulation relative to the standard full agonists DAMGO (MOP), DPDPE (DOP) or U69,593 (KOP). ^c^ not determined. ^d^ Determined in the [^35^S]GTPγS binding assay using rat brain membranes; percentage of stimulation relative to the standard full agonist DAMGO (MOP). ^e^ unpublished data. Data from [90,94,102]. For compound structures, refer to Scheme 1, Scheme 7 and Scheme 8.

**Table 7:** Antinociceptive potencies of differently 5-substituted *N*-methylmorphinan-6-ones **2**, **4**, **17** and **21**.

	ED_50_ (µg/kg, s.c.) ^a^		
	Hot-Plate Test	Tail-Flick Test	PPQ Abdominal Stretching Test
**2**	17	14	- ^b^
**4**	30	30	9.0
**17**	0.10	0.08	0.16
**21**	53	43	-
**Morphine**	850	1920	400

^a^ Determined in mice after s.c. administration. ^b^ not determined. Data from [90,102]. For compound structures, refer to Scheme 1, Scheme 7 and Scheme 8.

**Table 8:** Binding affinities at the opioid receptors of *N*-methylmorphinans **22**, **1**, **2** and **4**, and their *N*-phenethyl substituted analogues **22b**, **1b**, **2b** and **4b**.

	Binding Affiniy K_i_ (nM) ^a^			
	MOP	DOP	KOP	K_i_ Ratios MOP/DOP/KOP
Morphine (**22**)	3.35	195	96.4	1/58/29
**22b**	0.25	24.5	93.5	1/98/374
Oxymorphone (**1**)	1.41	79.1	32.6	1/56/23
**1b**	0.12	10.7	42.2	1/89/352
**2**	0.27	9.08	10.3	1/34/38
**2b**	0.19	1.81	15.8	1/9.5/83
**4**	0.25	18.6	12.8	1/74/51
**4b**	0.24	1.45	35.3	1/6.0/147

^a^ Determined in competition binding assays using membranes from CHO cells stably expressing human opioid receptors. Data from [94,116]. For compound structures, refer to Figure 3.

**Table 9:** In vitro agonist activities and antinociceptive of *N*-methylmorphinans **22**, **1**, **2** and **4**, and their *N*-phenethyl substituted analogues **22b**, **1b**, **2b** and **4b**.

	In Vitro Agonist Activity ^a^						Antinocieption ^b^
	MOP		DOP		KOP		ED_50_ mg/kg, s.c.
	EC_50_ (nM)	% stim.	EC_50_ (nM)	% stim.	EC_50_ (nM)	% stim.	
>Morphine (**22**)	34.4	89	668	109	710	76	3.06
**22b**	10.3	113	712	138	1049	19	0.11
Oxymorphone (**1**)	7.80	92	259	87	463	48	0.35
**1b**	2.67	97	131	101	225	7.5	0.15
**2**	1.21	95	38.5	102	135	65.9	0.014
**2b**	1.26	98	9.34	107	144	35.4	0.014
**4**	2.66	99	36.8	100	181	68.9	0.024
**4b**	1.86	102	9.54	103	334	51.3	0.024

^a^ Determined in the [^35^S]GTPγS binding assays using CHO stably expressing human opioid receptors; percentage stimulation relative to the standard full agonists DAMGO (MOP), DPDPE (DOP) or U69,593 (KOP). ^b^ Determined in the tail-flick test in mice after s.c. administration. Data from [94,116]. For compound structures, refer to Figure 3.
